# A three year descriptive study of early onset neonatal sepsis in a refugee population on the Thailand Myanmar border

**DOI:** 10.1186/1471-2334-13-601

**Published:** 2013-12-21

**Authors:** Claudia Turner, Paul Turner, Gabie Hoogenboom, Naw Aye Mya Thein, Rose McGready, Kawalee Phakaudom, Aruni De Zoysa, Androulla Efstratiou, Paul T Heath, François Nosten

**Affiliations:** 1 Shoklo Malaria Research Unit, Mae Sot 63110, Thailand; 2 Mahidol-Oxford Tropical Medicine Research Unit, Bangkok 10400, Thailand; 3 Centre for Tropical Medicine, University of Oxford, Oxford OX3 7LJ, United Kingdom; 4 Health Protection Agency, Microbiology Services Division, Colindale, London NW9 5EQ, United Kingdom; 5 St George’s, University of London, London SW17 0RE, United Kingdom

## Abstract

**Background:**

Each year an estimated four million neonates die, the majority in the first week of life. One of the major causes of death is sepsis. Proving the incidence and aetiology of neonatal sepsis is difficult, particularly in resource poor settings where the majority of the deaths occur.

**Methods:**

We conducted a three year observational study of clinically diagnosed early onset (<7 days of age) neonatal sepsis (EONS) in infants born to mothers following antenatal care at the Shoklo Malaria Research Unit clinic in Maela camp for displaced persons on the Thailand-Myanmar border. Episodes of EONS were identified using a clinical case definition. Conventional and molecular microbiological techniques were employed in order to determine underlying aetiology.

**Results:**

From April 2009 until April 2012, 187 infants had clinical signs of EONS, giving an incidence rate of 44.8 per 1000 live births (95% CI 38.7-51.5). One blood culture was positive for *Escherichia coli*, *E. coli* was detected in the cerebrospinal fluid specimen in this infant, and in an additional two infants, by PCR. Therefore, the incidence of bacteriologically proven EONS was 0.7 per 1000 live births (95% CI 0.1 – 2.1). No infants enrolled in study died as a direct result of EONS.

**Conclusion:**

A low incidence of bacteriologically proven EONS was seen in this study, despite a high incidence of clinically diagnosed EONS. The use of molecular diagnostics and nonspecific markers of infection need to be studied in resource poor settings to improve the diagnosis of EONS and rationalise antibiotic use.

## Background

Infections are the commonest cause of death in infants less than four weeks old (neonates) [1-3]. Every year an estimated four million neonates die; 99% of these deaths occur in the developing world with the majority in the first week of life [4-6].

Neonatal sepsis can be defined clinically and/or by positive microbiology from normally sterile site specimens (blood, cerebrospinal fluid (CSF) or urine obtained in a sterile manner). A diagnosis of neonatal sepsis based only on microbiological findings will under estimate the true burden of neonatal sepsis [7]. However, information regarding pathogen-specific disease incidence can only be derived from studies that rely on microbiological diagnosis. Unfortunately there are various reasons why microbiological confirmation may not be possible, particularly in the developing world. These include limited laboratory facilities, difficulty in obtaining a sufficient quantity of blood from a small infant and suppression of bacterial growth in cultures by antibiotics given in labour [8].

A recent review of the pathogens associated with infection in infants in the developing world reported that in infants less than seven days (early onset neonatal sepsis (EONS)) Gram negative organisms predominated, in a ratio of 2:1, with *Escherichia coli* being the most commonly isolated pathogen. The authors suggested that the reason that Gram negative organisms predominated in EONS is that they were environmentally acquired during unhygienic birth practices [9].

Historically, infection with GBS has been reported to be extremely rare in the developing world [1]. Explanations for this include less maternal carriage or less virulent strains of GBS, but also study design [10]. EONS GBS disease presents early in life with 90% of cases of sepsis presenting within 12 hours of birth [11,12]. Therefore, studies looking at EONS in community born infants presenting to hospitals will be biased, as infants with EONS GBS have a high likelihood of dying at home or on the way to the hospital. Additionally, a recent systematic review demonstrated that studies in which the use of intrapartum antibiotics (IAP) are reported are associated with lower rates of EO GBS disease [13]. More recent African studies have reported much higher rates of early onset neonatal GBS infection [14-18]. A review paper by Seale et al. concludes that these higher recorded incidences are due to more sensitive study designs that focus on infants born in hospital, who develop early onset sepsis, and not on outpatient referrals [19].

Mortality from neonatal sepsis, from any cause, is high, even when treated. Case fatality rates of 5 – 60% are reported with the highest mortality rates occurring in developing countries [6,20]. A large multicentre case controlled study performed in the USA between 1995 and 1996 showed an incidence of EONS of 3.5 per 1000 live births with 16% of these neonates dying [21].

The aims of the current study were to: (i) describe the epidemiology of clinically diagnosed EONS in a rural South East Asian refugee population and (ii) to attempt to determine the aetiology of EONS, in particular to ascertain whether GBS is an important pathogen in the area.

## Methods

### Study population

Maela is a crowded camp for displaced persons from Myanmar located on the north-western border of Thailand. Approximately 50,000 refugees live in a 4 km^2^ area and most refugees are from the Karen ethnic group. Shoklo Malaria Research Unit (SMRU) has been providing onsite medical and obstetric care in Maela since 1986. There are approximately 1,500 deliveries per annum in the camp and 90% of pregnant women attend SMRU’s antenatal clinics, with 80% of these women delivering at the SMRU obstetric unit. Deliveries are performed by skilled birth attendants. Complex obstetric cases and women requiring a caesarean section for delivery are transferred to the local Thai Hospital in Mae Sot (60 km away). Gestation of all infants born to mothers following antenatal care is routinely estimated either at the first antenatal consultation by USS or by Dubowitz gestational assessment after delivery [22,23]. Using population norms, an infant is determined to be small for gestational age (SGA) if their weight falls below the 10th centile for weight [24]. All neonatal care is undertaken in the Special Care Baby Unit (SBCU), located in the SMRU clinic. The pregnant population has a low rate of HIV and syphilis [25].

### Enrolment criteria

Over a three year period the mothers of all infants clinically diagnosed with EONS and admitted to the SCBU in Maela camp were asked to participate in the study. Informed written consent was obtained in a timely manner so as not to delay treatment of the infants. In order to determine the accurate mortality from EONS and to maximise the possibility of obtaining a positive blood culture, infants were excluded from being enrolled into the study if they had a severe congenital abnormality identified prenatally or at birth, or had received antibiotics within the early neonatal period (≤6 days of age).

We diagnosed EONS clinically using previously published criteria: an infant less than seven days of age with a fever (>38°C on one occasion or >37.5°C on two occasions separated by at least one hour) or at least two clinical features (poor perfusion, respiratory distress, persisting glucose imbalance, abdominal distension, bilious aspirates, or blood in the stool in a baby <72 hours of age) [26]. EONS could not be diagnosed using biochemical or haematological parameters, since the SMRU laboratory is located 60 km away.

### Specimen collection and laboratory testing

Once a diagnosis of EONS was made clinically and consent obtained, the following samples were collected from the infant: complete blood count (PocH-one 100i, Sysmex), C-reactive protein (NycoCard, Axis-Shield), blood culture and cerebrospinal fluid (CSF). A vaginal-rectal swab was obtained from the mother.

### Blood cultures

One millilitre of venous blood was collected into a BacT/ALERT PF culture bottle (bioMerieux). From 2nd April 2011 until the end of the study all blood culture bottles were weighed before and after blood collection to permit calculation of the volume of blood collected. The bottle was incubated at 37°C for up to five days in a BacT/ALERT 3D 60 instrument before being discarded as negative. Bottles with a positive growth signal were vented, examined by Gram stain, and sub-cultured onto appropriate agar plates (Clinical Diagnostics). Cultured organisms were identified by conventional microbiological techniques. Coagulase negative staphylococci, diphtheroids and *Bacillus* species were considered contaminants [27].

### CSF specimens

CSF specimens were examined on site and a cell count performed. The remaining specimen was sent to the SMRU microbiology laboratory for Gram stain and microbiological culture. Unfortunately protein estimation was unable to be performed as this investigation was not available in the SMRU laboratory. An aliquot of CSF was stored at -80°C for pathogen-specific PCR. Briefly, DNA was extracted from 200 μL of thawed CSF using an automated extraction protocol (Genomic DNA Whole Blood kit, MagCore HF16, RBC Bioscience). Multiplex PCR assays were used to detect *Chlamydia trachomatis*, *E. coli*, GBS, *Listeria monocytogenes*, *Staphylococcus aureus*, *Ureaplasma urealyticum*, cytomegalovirus, adenovirus, enteroviruses and parechoviruses following the manufacturer’s protocol (FTD Neonatal sepsis kit, Fast-track Diagnostics).

### Vaginal-rectal swabs

Vaginal-rectal swabs were collected and processed according to the CDC guideline for detection of GBS colonisation [28]. Briefly, swabs were cultured overnight in LIM broth and 10 μL of this broth was sub-cultured onto 5% sheep blood agar and incubated overnight in 5% CO_2_ at 36°C. Morphologically suspected GBS colonies were confirmed as described above. All GBS isolates were serotyped by latex agglutination (Strep-B-Latex kit, Statens Serum Institute). Those isolates that were non-typeable by this serotyping method or had a weak positive reaction were further characterised using multiplex PCR assays based on the detection of GBS capsular genotypes (Ia, Ib-IX) [29]. In addition to culture, an aliquot of LIM broth culture was used for detection of GBS by PCR, as previously described [30]. GBS capsular genotype was determined by for those specimens with a positive GBS PCR, by the same multiplex PCR assays used for cultured isolates.

### Clinical management

After the specimens were obtained the infant was commenced on antimicrobial treatment: intravenous ampicillin and gentamicin. A presumptive diagnosis of meningitis was made if the CSF WBC was above the upper limit of normal (30×10^9^/L [31]), in these cases intravenous cefotaxime was also given. The length of antibiotic treatment was determined on microbiological results. If the CSF was culture negative but the WBC was >50×10^9^/L, a diagnosis of meningitis was made and cefotaxime was continued for ≥10 days [27].

The infant was discharged from hospital when completely recovered and afebrile, was breast feeding well and gaining weight. The infant was then followed up at seven and 28 days of age to ensure that they were well and thriving.

### Data analysis

Data were entered into an Access 2003 database (Microsoft) and statistical analyses carried out using Stata/IC 12.1 (StataCorp). Student’s t-test was used to compare means and the two-sample Wilcoxon rank sum test was used to compare medians. The Chi-squared test was used to compare proportions, with two-tailed p-values of <0.05 indicating significance.

### Ethical approval

All women gave informed consent to participate in the study. Ethical approval was granted by the Ethics Committee of The Faculty of Tropical Medicine, Mahidol University, Thailand (MUTM 2009-011-03) and the Oxford Tropical Research Ethics Committee, Oxford University, UK (48–08).

## Results

**Figure 1:**
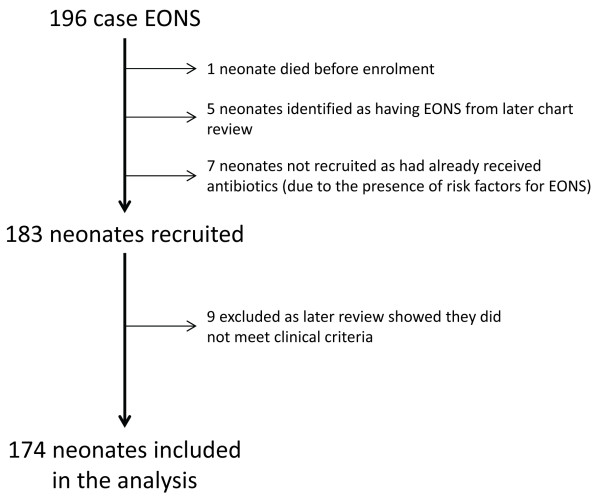
Summary of EONS cases from April 2009 until April 2012 in Maela Camp and enrolment into the study.

Between 20th April 2009 and 19th April 2012, 196 episodes of EONS were diagnosed. One neonate, born at home, died before he could be enrolled. Five further infants, who fulfilled the clinical criteria for a diagnosis of EONS were identified on subsequent clinical record review and seven infants with clinical signs of EONS could not be enrolled as they had already started on antibiotics (because of the presence of risk factors for EONS but not clinical features of sepsis). A total of 183 infants were recruited into the study. Nine (5%) of these infants were recruited in error as they did not meet the inclusion criteria. The remaining 174 infants are described further (Figure 1).

### Incidence of early onset neonatal sepsis

**Figure 2:**
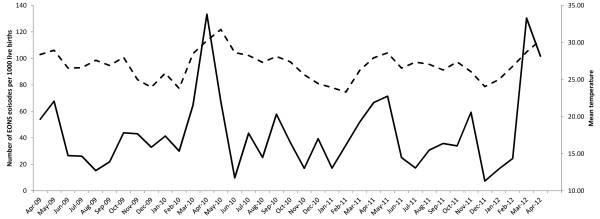
**Number of EONS episodes per 1000 live births, by month of diagnosis (primary axis; solid line).** Mean monthly temperature (secondary axis; dashed line).

There were 4,173 live births to women following antenatal care with SMRU over the study period, giving an incidence for clinically diagnosed early onset neonatal sepsis of 44.8 per 1,000 live births (187/4,173, 95% CI 38.7-51.5). There were peaks in the incidence of EONS in March and April each year, the hottest months of the year (Figure 2).

### Infant characteristics

Of the infants enrolled, 96/174 (55.2%) were male. The median gestation was 39^+^5 weeks (range 30^+^5 to 41^+^6); 10/174 (5.8%) of infants were born prematurely (>28 to 36 ^+^6 weeks). The median birth weight was 3.17 kg (range 1.42 – 5.08 kg), 12/172 (7.0%) were small for gestational age. The majority of the infants enrolled were born at the SMRU clinic, with few being born at home (5/174, 2.9%) or in Mae Sot hospital (3/174, 1.7%).

### Early onset neonatal sepsis episodes

The median age of admission to SCBU for clinically diagnosed EONS was one day (range 0 – 5 days). The commonest reason for enrolment into the study was a fever >38°C (Table 1).

**Table 1:** Clinical features of 174 neonates diagnosed with EONS and enrolled into the study

**Clinical feature**	Number of infants* (%)
Temperature	
>38°C	104 (59.8)
>37.5°C on two occasions separated by at least one hour	66 (37.9)
Poor perfusion	5 (2.9)
Respiratory distress	15 (8.6)
Persisting glucose imbalance	3 (1.7)
Abdominal distension	2 (1.2)
Bilious aspirates	3 (1.7)
Blood in the stool in a baby < 72 hours of age	1 (0.6)

*some babies had more than one feature hence total number is great than 174.

Only 5/174 (2.9%) infants had two or more clinical features of EONS and one of these infants also had a temperature >38°C. Of the infants with a temperature of >38°C, 8/104 (7.7%) also had other clinical signs of sepsis. Of the infants with a temperature >37.5°C on two occasions, 14/66 (21.2%) also had other clinical signs of sepsis. Therefore 147/174 (84.5%) of EONS cases were diagnosed by fever alone.

### Investigations

Blood cultures were positive in 40/171 cases and in 39 (22.8%) these were believed to be contaminants. *Escherichia coli* was identified from one blood culture (1/171; 0.3%).

The mean volume of blood sent for blood culture was 0.8 ml (SD 0.5 ml).

A total of 135 lumbar punctures were successfully performed (77.6% of all infants enrolled). Of these, 130 had a cell count performed and 13 (10.0%) had a WBC count >50×10^9^/L. There was a trend for a significantly higher CRP in these infants as compared to those who had a CSF WBC of <50×10^9^/L (p = 0.06). There were no clinically significant bacterial isolates cultured. One hundred and thirty two CSF samples underwent pathogen-specific PCR. *E. coli* DNA was found in three samples (one of which was from the infant in whom *E. coli* was cultured from blood). All three infants were term and of normal birth weight. All presented before 72 hours of age with a fever and no other clinical signs of sepsis. Two of the mothers had prolonged rupture of membranes (> 18 hours, PROM) and were treated with antibiotics during labour (one with ampicillin and one with amoxicillin), the third had no documented prior antibiotic exposure. All three of the infants had a CRP taken, the neonate with the positive blood culture had the highest value (46 mg/L) and the other two had CRP values of 8 and 12 mg/L. None of these infants had a raised WBC in their CSF, with the CSF white cell counts being 6, 4 and 2×10^9^/L.

### Maternal vaginal rectal swabs

GBS was cultured from 4/170 (2.4%) maternal vaginal swabs and a further 11 swabs were GBS positive by PCR, giving an overall carriage rate of 8.8% (95% CI 5.0 – 14.2). This prevalence is not significantly different to that found in a GBS carriage study described previously in this population (12.0%; p = 0.3) [30]. None of the mothers who had a vaginal swab positive for GBS by culture received antibiotics in labour while 2/11 (18.2%) of the GBS positive by PCR had received antibiotics during labour. Serotype III was the most common GBS serotype identified by culture and serotype Ib was the most common serotype detected by PCR.

### Complete blood count

The median WBC value was 13.6 with a range of 5.4 – 38.0×10^6^/L. No infants with EONS had a WBC of <5.0×10^6^/L but nine infants (5.3%) had a raised WBC (>26.0×10^6^/L [32]. Of the infants with EONS, 18/171 (10.5%) had a platelet count of <150 × 10^9^/L. Of the infants who were diagnosed on clinical signs alone (i.e. no fever), 4/5 (80%) had a low platelet count (p < 0.0001).

### C-reactive protein

C-reactive protein results ranged from the lowest limit of detection (8 mg/L) to 109 mg/L; 86/165 (52.1%) had a CRP >8 mg/L.

### Maternal characteristics

The median age of the mothers was 26.5 years (range 16.0 – 44.0 years); 7/174 (4.0%) of the mothers were <18 years old. The median gravidity of these women was two (range 1 – 10) and parity two (range 1 – 10). Fourteen (8.1%) mothers had a previous neonatal death, ten of which occurred in the early neonatal period. The causes of death were generally unknown but three reported that their infant was born prematurely and one that their infant died of jaundice. Four of these infants died at home without their parents seeking medical care.

### Labour

The median length of rupture of membranes (ROM) was 3.1 hours (range 0 minutes – 17 days). Twenty nine (29/171, 17.0%) women had PROM (ROM >18 hours). This was significantly higher than the proportion of women who had PROM in a previously published study on GBS carriage, in the same population (17.0% versus 7.3% p < 0.001 [30]). Overall 33/171 (19.3%) women received antibiotics during labour. Of the women with PROM, 28/29 (97%) received antibiotics in labour. Twenty six (26/28, 93.0%) received ampicillin, 1/28 (3.6%) received amoxicillin and 1/28 (3.6%) received ceftriaxone. Four other women received antibiotics in labour: three received ampicillin (two for chorioamnionitis and one for pyelonephritis) and one ciprofloxacin (for pyelonephritis). The median interval between antibiotic administration and delivery was nine hours (range 0 – 36 hours). Nine women had a documented fever during labour and four of these women received antibiotics.

### Outcomes

The median length of hospital admission was five days but the range was large, from 2 – 46 days. There were two (1.1%) early neonatal deaths in the study infants; however neither death was directly related to the sepsis episode. One infant had Down’s syndrome and a congenital cardiac malformation and one was born at 30 + 5 weeks gestation and died of clinically diagnosed respiratory distress syndrome.

## Discussion

This three year observational study identified neonates who had clinical features of EONS and were born to mothers who had antenatal care in a refugee camp on the Thailand-Myanmar border. The objective of the study was to describe the epidemiology of clinically diagnosed EONS and to investigate possible bacterial causes and in particular GBS.

The incidence of EONS using a clinical definition was 44.8 episodes per 1,000 live births. It is not possible to compare this figure to that of other studies as clinically diagnosed EONS is not usually reported. This is unfortunate as we recognise that the true burden of neonatal sepsis will be greater than that which is simply based on positive blood cultures.

The majority of infants were diagnosed with EONS because they had a fever. Only four infants were diagnosed as having EONS because of the presence of other clinical signs. All temperatures were taken rectally and therefore accurately reflect core body temperature. Although correctly taking a rectal temperature does require training it is more straight forward to teach and to ensure accuracy (as the outcome is objective and not subjective) than the recognition of clinical signs. Hence it may be reasonable to use only the presence of fever as a diagnostic criterion for EONS in health care facilities run by staff with limited training. The incidence of EONS increased in March – May, corresponding to the hot season. This begs the question as to whether the infants who were diagnosed with EONS because of a fever in this time period, had a fever because of the high ambient temperature and not because they had true EONS.

Using clinical features alone may overestimate the incidence of EONS leading to the overuse of antibiotics. In our study only 86/165 (52.1%) infants who were clinically diagnosed as having EONS had a raised CRP at presentation. The National Institute for Health and Clinical Excellence has published guidelines for the use of antibiotics for EONS [33]. These guidelines recommend starting antibiotics in any neonate who has clinical signs of sepsis but to repeat a CRP measurement 18 – 24 hours after starting treatment, to determine those infants who truly do have sepsis. Adopting this strategy would increase the accuracy of calculating the incidence of neonatal sepsis in an environment where microbiological diagnosis is not possible.

The incidence of bacteriologically proven EONS was 0.7 per 1,000 live births (95% CI 0.1 – 2.1). This incidence is lower than that reported from other developing country settings. For example, in Kenya, Berkley et al. report a minimal rate for neonatal bacteraemia of 5.5 per 1,000 live births [14]. It was also notable that a significant proportion (10%) of infants who had an LP performed were found to have a raised CSF white cell count (>50 cells/mm^3^). Using a “normal” definition of < 20 white cells [34] increases this proportion to 30%, yet a pathogen was not detected in any of these samples.

*Escherichia coli* was the only pathogen isolated from a blood culture and the only pathogen identified in CSF. The mothers of two of the three infants with a positive CSF *E. coli* PCR had received antibiotics in labour, potentially explaining the lack of bacterial growth. Specific PCRs were not available for all potentially relevant bacteria (e.g. *Klebsiella* spp.), thus we cannot exclude the possibility that other bacteria were implicated.

One explanation for the lack of positive microbiology is the high quality antenatal care that women living in Maela camp receive and the high rate of deliveries with skilled birth attendants, resulting in prompt antibiotic administration during labour when necessary. A significant proportion of women whose babies subsequently had clinical sepsis had prolonged rupture of membranes and 97% of these women had received antibiotics in labour.

One other significant factor is the use of un-prescribed antibiotic use in the community. Antimicrobials are readily available for purchase at many shops within Maela. A survey conducted in 2011 found that 25% of pregnant women had evidence of antimicrobial activity in their urine suggesting recent antimicrobial consumption (unpublished data). Maternal antibiotic use could suppress bacterial growth in samples collected from neonates, decreasing the conventional microbiological utility in determining aetiology.

It is conceivable that the use of specific PCRs against a larger number of potential pathogens (e.g. *Klebsiella* spp., additional viruses, etc.) might increase the yield of the CSF samples. Further work should be focused on this apparent burden of infection.

A further possible explanation for the low bacteraemia incidence is the amount of blood collected for each blood culture sample and the high bacterial contamination rate. However both values were similar to that reported by Berkley et al. [14].

GBS is increasingly being reporting as a significant pathogen in the developing world, particularly in Southern Africa [15,18]. However GBS is unlikely to be an important pathogen in the current study described here. One explanation is that the proportion of women found to be colonised with GBS was relatively low. Additionally, there was significant use of IAP in this population, with nearly 20% of all women receiving IAP and 76.9% of women with significant risk factors for EOGBS (PROM, premature delivery and fever) receiving IAP. It is therefore conceivable that these antibiotics suppressed the growth of GBS in conventional cultures resulting in clinical disease with negative cultures. This suggests a potential role for molecular diagnostics and, although a GBS-specific PCR was possible for CSF samples (yielding no cases of GBS meningitis), it was not possible to do this on blood samples. The burden of EOGBS may therefore still be underestimated. It is also conceivable that the use of IPA could delay the onset of sepsis, however in the population described in this manuscript no neonate developed late onset sepsis from 7 – 28 days of age).

The majority of infants enrolled into the study were born at SMRU’s clinic. It has been argued that inborn infants are often over represented in studies on EONS as neonates who are born at home and develop EONS may die before reaching a health care facility [19]. This was not the case with our study, as all infants born to mothers who followed ANC at SMRU Maela were reviewed at 28 days of age in order to determine the neonatal mortality rate of the population. No infant was identified as having died from a potential septic episode at home.

No infant who was enrolled in this study died as a direct result of EONS. The only death from EONS that occurred at SMRU during the study period was in 2009, before the infant could be enrolled into the study. In total there were 55 early neonatal deaths over the study period. Using Lawn’s estimation that 26% of neonatal deaths are caused by sepsis, we could have expected to see 14 neonatal deaths due to EONS in this population in the time period studied. The explanation for this is multifactorial. Women received antibiotics when presenting with PROM and this may have reduced the infant’s risk of developing EONS. Additionally, medical staff were trained in the recognition of EONS and management protocols were clear.

## Conclusion

In conclusion, we have presented a three year study defining the epidemiology of EONS in a SE Asian refugee population. *E. coli* was the only bacteria isolated and GBS was not found to be the cause of any EONS episodes. The lack of bacterial isolates from conventional microbiological culture, potentially due to high antibiotic use in the community (both medically prescribed and self-administered), highlights a problem common to many resource poor settings. The use of nonspecific markers of infection should be examined in these settings in order to make more accurate diagnosis of sepsis in infants.

We postulate that rigorous use of locally appropriate guidelines (including IAP), obstetric and neonatal, a high proportion of deliveries with skilled birth attendants, regular training and motivated staff, resulted in the absence of EONS-attributable mortality in infants born in Maela.

## Competing interests

The authors declare that they have no competing interests.

## Authors’ contributions

CT, PT, PH and FN conceived the study. AMT, GH, RM and CT were responsible for specimen and data collection. KP, AE, ADZ and PT performed the laboratory work. CT did the data analysis and prepared the first draft of the manuscript. All authors reviewed and contributed to revisions of the manuscript. All authors read and approved the final manuscript.

## Pre-publication history

The pre-publication history for this paper can be accessed here:

http://www.biomedcentral.com/1471-2334/13/601/prepub

## References

[B1] Vergnano S Sharland M Kazembe P Mwansambo C Heath PT Neonatal sepsis: an international perspective Arch Dis Child Fetal Neonatal Ed 2005 13 3 F220 F224 10.1136/adc.2002.022863 15846011 PMC1721871

[B2] Stoll BJ The global impact of neonatal infection Clin Perinatology 1997 13 1 1 21 9099499

[B3] Qazi SA Stoll BJ Neonatal sepsis: a major global public health challenge Pediatric Infect Dis J 2009 13 1 Suppl S1 S2 10.1097/INF.0b013e31819587a9 19106756

[B4] Zupan J Perinatal mortality for the year 2000: estimates developed by WHO 2005 Geneva: WHO

[B5] Lawn JE Cousens S Zupan J 4 million neonatal deaths: when? Where? Why? Lancet 2005 13 9462 891 900 10.1016/S0140-6736(05)71048-5 15752534

[B6] Thaver D Zaidi AK Burden of neonatal infections in developing countries: a review of evidence from community-based studies Pediatric Infect Dis J 2009 13 1 Suppl S3 S9 10.1097/INF.0b013e3181958755 19106760

[B7] Gessner BD Castrodale L Soriano-Gabarro M Aetiologies and risk factors for neonatal sepsis and pneumonia mortality among Alaskan infants Epidemiol Infect 2005 13 5 877 881 10.1017/S0950268805004449 16181508 PMC2870319

[B8] Heath PT Schuchat A Perinatal group B streptococcal disease Best Pract Res Clin Obstet Gynaecol 2007 13 3 411 424 10.1016/j.bpobgyn.2007.01.003 17336588

[B9] Zaidi AK Thaver D Ali SA Khan TA Pathogens associated with sepsis in newborns and young infants in developing countries Pediatric Infect Dis J 2009 13 1 Suppl S10 S18 10.1097/INF.0b013e3181958769 19106757

[B10] Howard JB McCracken GH Jr The spectrum of group B streptococcal infections in infancy Am J Dis Child 1974 13 6 815 818 4613165 10.1001/archpedi.1974.02110310063011

[B11] Airede AI Neonatal septicaemia in an African city of high altitude J Trop Pediatr 1992 13 4 189 191 10.1093/tropej/38.4.189 1527816

[B12] Quiambao BP Simoes EA Ladesma EA Gozum LS Lupisan SP Sombrero LT Romano V Ruutu PJ Serious community-acquired neonatal infections in rural Southeast Asia (Bohol Island, Philippines) J Perinatol: official journal of the California Perinatal Association 2007 13 2 112 119 10.1038/sj.jp.7211633 17262044

[B13] Edmond KM Kortsalioudaki C Scott S Schrag SJ Zaidi AK Cousens S Heath PT Group B streptococcal disease in infants aged younger than 3 months: systematic review and meta-analysis Lancet 2012 13 9815 547 556 10.1016/S0140-6736(11)61651-6 22226047

[B14] Berkley JA Lowe BS Mwangi I Williams T Bauni E Mwarumba S Ngetsa C Slack MP Njenga S Hart CA Bacteremia among children admitted to a rural hospital in Kenya N Engl J Med 2005 13 1 39 47 10.1056/NEJMoa040275 15635111

[B15] Gray KJ Bennett SL French N Phiri AJ Graham SM Invasive group B streptococcal infection in infants, Malawi Emerg Infect Dis 2007 13 2 223 229 10.3201/eid1302.060680 17479883 PMC2725867

[B16] English M Ngama M Musumba C Wamola B Bwika J Mohammed S Ahmed M Mwarumba S Ouma B McHugh K Causes and outcome of young infant admissions to a Kenyan district hospital Arch Dis Child 2003 13 5 438 443 10.1136/adc.88.5.438 12716721 PMC1719579

[B17] Milledge J Calis JC Graham SM Phiri A Wilson LK Soko D Mbvwinji M Walsh AL Rogerson SR Molyneux ME Aetiology of neonatal sepsis in Blantyre, Malawi: 1996–2001 Ann Trop Paediatr 2005 13 2 101 110 10.1179/146532805X45692 15949198

[B18] Madhi SA Radebe K Crewe-Brown H Frasch CE Arakere G Mokhachane M Kimura A High burden of invasive *Streptococcus agalactiae* disease in South African infants Ann Trop Paediatr 2003 13 1 15 23 10.1179/000349803125002814 12648320

[B19] Seale AC Mwaniki M Newton CR Berkley JA Maternal and early onset neonatal bacterial sepsis: burden and strategies for prevention in sub-Saharan Africa Lancet Infect Dis 2009 13 7 428 438 10.1016/S1473-3099(09)70172-0 19555902 PMC2856817

[B20] Edmond K Zaidi A New approaches to preventing, diagnosing, and treating neonatal sepsis PLoS medicine 2010 13 3 e1000213 10.1371/journal.pmed.1000213 20231868 PMC2834705

[B21] Schuchat A Zywicki SS Dinsmoor MJ Mercer B Romaguera J O’Sullivan MJ Patel D Peters MT Stoll B Levine OS Risk factors and opportunities for prevention of early-onset neonatal sepsis: a multicenter case–control study Pediatrics 2000 13 1 Pt 1 21 26 10617699 10.1542/peds.105.1.21

[B22] Rijken MJ Lee SJ Boel ME Papageorghiou AT Visser GH Dwell SL Kennedy SH Singhasivanon P White NJ Nosten F Obstetric ultrasound scanning by local health workers in a refugee camp on the Thai-Burmese border Ultrasound Obstet Gynecol : the official journal of the International Society of Ultrasound in Obstetrics and Gynecology 2009 13 4 395 403 10.1002/uog.7350 PMC3438883 19790099

[B23] Dubowitz LM Dubowitz V Goldberg C Clinical assessment of gestational age in the newborn infant J Pediatr 1970 13 1 1 10 10.1016/S0022-3476(70)80038-5 5430794

[B24] Rijken MJ Malaria in pregnancy: ultrasound studies of fetal growth 2012

[B25] Plewes K Lee T Kajeechewa L Thwin MM Lee SJ Carrara VI Nosten F McGready R Low seroprevalence of HIV and syphilis in pregnant women in refugee camps on the Thai-Burma border Int J STD AIDS 2008 13 12 833 837 10.1258/ijsa.2008.008034 19050214

[B26] Russell B Confirmed group B streptococcus infection: the tip of the iceberg Arch Dis Child Fetal Neonatal Ed 2001 13 84 F140 10.1136/fn.84.2.F140b PMC1721236 11330249

[B27] Talbert AW Mwaniki M Mwarumba S Newton CR Berkley JA Invasive bacterial infections in neonates and young infants born outside hospital admitted to a rural hospital in Kenya Pediatr Infect Dis J 2010 13 10 945 949 10.1097/INF.0b013e3181dfca8c 20418799 PMC3405819

[B28] Verani JR McGee L Schrag SJ Prevention of perinatal group B streptococcal disease--revised guidelines from CDC MMWR Recomm Rep 2010 13 RR-10 1 36 21088663

[B29] Afshar B Broughton K Creti R Decheva A Hufnagel M Kriz P Lambertsen L Lovgren M Melin P Orefici G International external quality assurance for laboratory identification and typing of *Streptococcus agalactiae* (Group B streptococci) J Clin Microbiol 2011 13 4 1475 1482 10.1128/JCM.02365-10 21325542 PMC3122801

[B30] Turner C Turner P Po L Maner N De Zoysa A Afshar B Efstratiou A Heath PT Nosten F Group B streptococcal carriage, serotype distribution and antibiotic susceptibilities in pregnant women at the time of delivery in a refugee population on the Thai - Myanmar border BMC Infect Dis 2012 13 1 34 10.1186/1471-2334-12-34 22316399 PMC3315410

[B31] Mandell M Principles and Pactice of Infectious Diseases, vol. 1 2010 7 Churchill Livingstone: Douglas and Bennette’s

[B32] Neil McIntosh PH Rosalind S Stuart L Forfar and Arneil’s Textbook of pediatrics 2008 7 Churchill Livingston: Churchill Livingstone

[B33] NICE Antibiotics for early-onset neonatal infection 2012 “NICE” National Institute for health and excellence

[B34] Kestenbaum LA Ebberson J Zorc JJ Hodinka RL Shah SS Defining cerebrospinal fluid white blood cell count reference values in neonates and young infants Pediatrics 2010 13 2 257 264 10.1542/peds.2009-1181 20064869 PMC3033868

